# Why communities must be at the centre of the Coronavirus disease 2019 response: Lessons from Ebola and human immunodeficiency virus in Africa

**DOI:** 10.4102/phcfm.v12i1.2496

**Published:** 2020-06-22

**Authors:** Oliver Johnson, Tinashe Goronga

**Affiliations:** 1 Department of Health Services and Population Research, Institute of Psychiatry, Psychology and Neuroscience, King’s College London, London, United Kingdom; 2 Centre for Health Policy, School of Public Health, University of the Witwatersrand, Johannesburg, South Africa; 3 Center for Health Equity Zimbabwe, Social Medicine Consortium Global Campaign Against Racism, Harare, Zimbabwe

**Keywords:** COVID-19, coronavirus, outbreak, non-pharmaceutical intervention, community, community engagement, empathy, behaviour change

## Abstract

As the Coronavirus disease 2019 (COVID-19) pandemic has spread globally, with no effective treatment or vaccine yet available, governments in many countries have put in place social interventions to control the outbreak. The various lockdown measures may have devastating impacts on economies and livelihoods. This approach risks undermining public trust in government responses and therefore undermines efforts to promote behaviour change, which is key to the success of social interventions. Important lessons can be drawn from past Ebola outbreaks and the human immunodeficiency virus pandemic on how communities should be central to COVID-19 responses. Communities are complex and only their members can inform public health experts about their lived realities, the community’s understanding of the outbreak and what will work locally to reduce transmission. The public should be encouraged to take positive actions to ensure their own health and well-being, rather than made to feel powerless. Communities should be supported to develop their own response plans, community leaders should be recognised as vital assets, community representatives should have equal inclusion in strategic meetings and greater empathy should be built into decision-making processes.

For social interventions to be sustainable, behaviour change at community level is essential. Communities must therefore be at the centre of the response to the Coronavirus disease 2019 (COVID-19) pandemic. There are currently no medical interventions available that will meaningfully curb the transmission of the virus, with effective treatments and vaccines months away. Reliance on social or ‘non-pharmaceutical interventions’ to bring the COVID-19 pandemic under control, including border closures, lockdowns and mandatory quarantining, may slow down the transmission but not eliminate it.^1^ Second and third surges of cases are probable as countries lift restrictions and businesses open up.^2^ Whilst a traditional approach to outbreak control is containment and eradication, we must learn to live with this virus and instead focus on suppression and mitigation.

From our experience of working in infectious disease control in Sierra Leone and Zimbabwe,^1^ we are convinced that responses must be done with communities, and not done to communities. Communities are complex with different social, religious, cultural and political dynamics that influence the uptake of health interventions. The Mayor of Freetown, Yvonne Aki-Sawyerr, emphasised during the Ebola crisis that consultation and engagement with communities were not enough; community ownership is essential. In the early stages of Sierra Leone’s Ebola outbreak, there was a strong emphasis on top-down decision-making and instructing communities what to do. Response leaders complained of ‘community resistance’ without recognising that people were just trying to survive. The prevalent judgement was that citizens could not be trusted to collaborate with the response. Punitive quarantine measures backfired, creating instead incentives to hide or flee from authorities rather than to cooperate, thereby driving cases underground. Dr Tom Frieden, Director of the United States Centers for Disease Control and Prevention (US-CDC) during Ebola, commented: ‘[*t*]he quarantine policy most likely prolonged the outbreak in Sierra Leone by at least six months (p. 342)’.^3^

The same mistakes are being repeated today with the COVID-19 pandemic. The essence of a lockdown is to deprive citizens of their freedom and their agency which risks treating them like prisoners or children, fuelling sentiments of powerlessness, resentment and fatalism, which do little to change behaviour. The language and approach to COVID-19 responses, in Africa and globally, has been of control and ‘enforcement’, often by police or military forces and sometimes involving violence.^4^ Lockdowns have disrupted people’s daily wages and livelihoods, threatening their survival, and undermining trust and confidence in their governments. Failing to address people’s legitimate concerns will promote more resistance, as witnessed in demonstrations by informal vendors in Malawi.^5^

Our work with HIV, sexual and reproductive health (SRH) and Ebola has taught us that nobody understands or cares more about health within a community than the community itself. Community members must be facilitated to educate public health experts about the lived realities of their lives, the feasibility of response plans and the perceptions and concerns that shape behaviour in their neighbourhoods. Of course, public health experts must provide accessible health messages about the transmission of COVID-19. Underlying cultural realities will only be unearthed when the community develops trust and confidence in health workers and response systems.

Calls for greater community engagement and social mobilisation are usually supported but rarely prioritised. With COVID-19, as with Ebola, immediate challenges like shortages of personal protective equipment, beds and testing kits seem so pressing that we feel we do not have time to do community engagement. The truth is that we do not have time NOT to do it. A colleague described with Ebola how ‘… social mobilisation would be twenty-ninth on the agenda at meetings’.^2^ We can start by moving it to the top.

Our experience of partnerships in HIV work between government facilities and civil society organisations in urban and rural areas of Zimbabwe demonstrated that these filled critical gaps, especially in gaining access to hard-to-reach or marginalised groups. Communities of people living with HIV (PWHIV), through peer education and advocacy, have been at the forefront of defending their right to health and access to essential services. People living with HIV groups taught us as health professionals how to overcome stigma and to deal with our fears of becoming infected. We can extend what we have learnt from PWHIV groups to support communities to feel more control over their health through positive actions which they can take to keep themselves and their families safe. Making cloth masks to wear in public, promotion of hand hygiene and collective provision of water and soap supplies, and provision of food parcels are some examples of community-based activities. These models of partnerships also provide models for work with communities that encourage support and recognition of health workers for their courage and sacrifice, and where survivors are embraced and cared for by their communities when they return home rather than being feared as carriers of infection.

Working as HIV and SRH campaigners demonstrated for us and others the importance of building social capital, trust and community cohesion to improve the effectiveness of health messages.^6^ A critical element with HIV and Ebola advocacy was working with traditional and community leadership, key people who are trusted in community networks. These are community assets: religious leaders, business people, politicians, traditional leaders and market sellers’ associations. It is important to establish partnerships with them, listen to their ideas, understand their fears, concerns and views on the COVID-19 outbreak, and then work together on a plan to keep everyone safe. Non-medical people should be recruited into the effort to contribute as community COVID-19 experts, similar to village health workers supporting primary care systems in rural areas.

We must build empathy into all our decision-making. For every policy decision, from large to small, we should pause and ask ourselves the question: how would I feel if this were me, or my child? For this to work, we should ensure that community leaders or representatives are invited to join our meetings and discussions, with equal status alongside clinicians and epidemiologists, and that their voices are sought and listened to. If this is missing, we can designate someone at a meeting to specifically reflect the interests and realities of the community in the conversation. Where it is not feasible to entirely stop a community activity that may pose a transmission risk, we should collaborate with the people affected to think through safer approaches rather than impose interventions on them. For example, busy street markets can be moved to large open spaces where traders can be safely separated from each other, with no contact policy between customer and seller and demarcated queues. Shielding of elderly family members may require changes in sleeping arrangements in rural homes, separate from schoolgoing children. Collaboration and co-developing new systems with affected communities may help in earning their trust and confidence rather than imposing measures on them.

Public health strategies should invest more in community ownership and participation instead of imposing interventions on people and punishing those who do not comply. With the current absence of medical treatment and vaccination, the pandemic can only be brought under control by massive and rapid behaviour change. This can help turn the tide and should be prioritised. The change in mindset will also help build foundations for improved public health and community partnerships towards a more community-oriented health system.

## Footnotes

1 O.J. has worked at the clinical and strategic levels of the 2014–2016 Ebola response in Sierra Leone for 18 months and co-wrote a book about the lessons from the crisis that drew on the experiences of survivors, frontline health workers and response leaders.

2 T.G. has worked in urban and rural hospitals in Zimbabwe as a government medical officer. He is a campaigner for human rights in sexual and reproductive health including HIV.
